# Strategies to Genetically Modulate Dendritic Cells to Potentiate Anti-Tumor Responses in Hematologic Malignancies

**DOI:** 10.3389/fimmu.2018.00982

**Published:** 2018-05-18

**Authors:** Annelisa M. Cornel, Niek P. van Til, Jaap Jan Boelens, Stefan Nierkens

**Affiliations:** ^1^Laboratory of Translational Immunology, University Medical Center Utrecht, Utrecht, Netherlands; ^2^Pediatric Blood and Marrow Transplantation Program, University Medical Center Utrecht, Utrecht, Netherlands; ^3^Blood and Marrow Transplantation Program, Princess Maxima Center for Pediatric Oncology, Utrecht, Netherlands

**Keywords:** dendritic cell, vaccination, genetic modification, hematopoietic cells, hematopoietic cell transplantation, cord blood

## Abstract

Dendritic cell (DC) vaccination has been investigated as a potential strategy to target hematologic malignancies, while generating sustained immunological responses to control potential future relapse. Nonetheless, few clinical trials have shown robust long-term efficacy. It has been suggested that a combination of surmountable shortcomings, such as selection of utilized DC subsets, DC loading and maturation strategies, as well as tumor-induced immunosuppression may be targeted to maximize anti-tumor responses of DC vaccines. Generation of DC from CD34+ hematopoietic stem and progenitor cells (HSPCs) may provide potential in patients undergoing allogeneic HSPC transplantations for hematologic malignancies. CD34+ HSPC from the graft can be genetically modified to optimize antigen presentation and to provide sufficient T cell stimulatory signals. We here describe beneficial (gene)-modifications that can be implemented in various processes in T cell activation by DC, among which major histocompatibility complex (MHC) class I and MHC class II presentation, DC maturation and migration, cross-presentation, co-stimulation, and immunosuppression to improve anti-tumor responses.

## Introduction

Although the overall survival rates of patients with hematologic malignancies have significantly increased in the past decades, the 5-year survival of certain acute leukemias, such as acute myeloid leukemia (AML) is still unsatisfactory due to high relapse risk (1–4). Currently, the only curative treatment consists of intense chemotherapy followed by hematopoietic cell transplantation (HCT), but only about 30% of candidates eligible for HCT transplantation have a human leukocyte antigen (HLA)-identical sibling as a donor for matched transplantation. Alternatively, bone marrow from unrelated volunteer donors could be used; however, this is limited by strict HLA-matching criteria, because of higher risks of graft-versus-host disease (GVHD), and donor availability.

Umbilical cord blood (UCB) transplantation has advantages because of its prompt availability from UCB banks, the possibility of HLA-mismatched transplantations, a lower risk of acute and chronic GVHD, and a potential higher graft-versus-leukemia effect (5–8).

Individualized dosing and timing of chemo and/or serotherapy improves overall survival of transplanted patients with hematologic malignancies after cord blood transplantation (9, 10). Cord blood T cells have shown the ability to rapidly reconstitute the immune system (9), and can mediate enhanced anti-tumor effects when compared with adult peripheral T cells (11). In addition, cord blood CD8+ T cells have shown to exhibit stronger proliferation potential and function after antigen-specific stimulation (12). The relatively low survival rate of patients with hematologic malignancies underlines the relevance to investigate novel potential effective therapies in the context of UCB transplantation to treat AML or other hematologic malignancies.

Tumor-associated antigen (TAA)-specific immunotherapy to prime the TAA-specific T cells against the leukemia to consequently induce remission has been thoroughly investigated. Four decades of research revealed the central role of dendritic cells (DCs) as a link between innate and adaptive immunity, and thereby its essential role in the control of both immune tolerance and immunity (13). The antigen presentation machinery of DCs is exploited in cellular vaccination strategies to initiate an endogenous anti-tumor response (14). The rationale for this approach is the generation of TAA-specific cytotoxic T lymphocyte (CTL) responses to specifically eradicate tumor cells and to generate immunological memory to control potential future tumor relapse (15). However, DC vaccine trials have only sporadically shown clinical responses. Insufficient DC maturation, suboptimal antigen presentation, co-stimulation, migration, or impaired initiation of anti-TAA T cell responses could be inherent to the cultured DC subset, but may also be influenced by the inhibition of immune responses by the tumor microenvironment (14, 16). Hence, efficacy of DC vaccination strategies can be improved by state-of-the-art genetic modification tools, such as messenger RNA, adenoviral and lentiviral vectors, and gene-editing techniques to enhance processes in DC activation of T cells (15, 17) and consequently boost immune responses. In this review, we will address modification of phenotypes and function of DCs, including cord blood CD34-derived DCs, to optimize the anti-tumor response to protect for relapses after HCT.

## DC Subsets Eligible for Modification

Although thoroughly investigated, there is still no consensus about the most optimal DC subset to use to induce optimal TAA-specific T cell responses (18). Circulating peripheral blood DCs are difficult to isolate, hence monocyte-derived DCs (moDCs), generated from peripheral blood mononuclear cells are the most commonly used. These cells are generated from monocytes by use of granulocyte macrophage colony stimulating factor and interleukin (IL)-4 (18). Although moDC-derived vaccines are reported to be safe, clinical responses have only sporadically been observed (15, 17).

Research investigating different DC subsets pointed to differential subsets (such as conventional and plasmacytoid DCs) and functionalities (19), which could be of importance to induce favorable immune responses. The advantage of using primary DCs is that they can be promptly isolated from blood, avoiding long differentiation incubation periods before administration to the patient, thereby making this strategy suitable for standardization for multicentre trials (20). However, the differentiated status of these cells is also a drawback, as this limits expansion of the cell population. As a result, large numbers of primary DC may be required to provide effective therapeutic dosing.

Another commonly used approach is to produce DCs from CD34+ hematopoietic stem and progenitor cells (HSPCs) (21, 22), which have an extensive proliferation capacity to generate antigen presenting cells (APCs) with a primary DC phenotype (23) and the capacity to induce robust anti-tumor T cell responses. These cells are distinct from moDCs (24–27), and more resemble conventional DC or Langerhans resembling cells (28) that induce stronger anti-tumor T cell responses compared with moDCs (29). In the setting of cord blood transplantation after chemotherapy in hematologic malignancies, CD34+ HSPCs can be extracted from 20% of the remaining unit that is not transplanted, and developed into an effective DC vaccine, that can be modified at different stages of the manufacturing process, which will be discussed below.

Vaccination with UCB CD34-derived DCs has been performed in clinical trials to treat patients with melanoma and showed TAA-specific responses in some patients (23, 30). The *ex vivo* culturing phase to generate CD34-derived DCs provides a unique opportunity to enhance efficacy through genetic modification. Principally, the expansion phase of the protocol could be extended to 2 weeks and this does this not affect DC maturation (26). This indicates that this two-step protocol allows opportunities to modify the CD34-derived DCs at the early stage as well as during the later stages of the protocol, as compared with DCs generated from other precursor subsets.

## Modulating TAA-Loading and Major Histocompatibility Complex (MHC)-I Presentation to Enhance DC Efficiency

Tumor-associated antigens are ideally over expressed on malignant cells and are simultaneously not expressed on healthy tissues or contain mutations leading to neo-antigens recognizable to T cells. Hence, a commonly used TAA is the oncoprotein Wilms’ tumor-1 (WT1), which has been ranked the number one cancer vaccine target antigen (31). WT1 is a zinc finger transcription factor with a well-established oncogenic role in WT1 overexpressing malignancies (32). WT1 overexpression is observed in the majority of acute leukemias (~90% of pediatric AML cases), as well as various solid tumors (33), making WT1 an obvious vaccine target. Despite its physiological expression in hematopoietic tissue–limited expression in the urogenital–and central nervous system (34), it has been shown that tumor overexpression of WT1 can be targeted without considerable safety concerns (35, 36). Several recent early-phase anti-WT1 DC vaccine clinical trials in multiple cancer types reported a correlation between anti-WT1 CTL responses and clinical response (35, 37, 38), showing its potential as a therapeutic strategy.

The most commonly used methods to present antigen are delivery of peptide pools or mRNA to express the tumor antigen-target, which result in the ability to transiently load DCs with antigen. An advantage to deliver mRNA is that it prevents HLA-restrictions and invasive tumor tissue isolation from patients. Alternatively, full-length WT1 mRNA can also be combined with a WT1 peptide pool to enhance its potential (14, 39). Two main modification strategies have been reported to potentially optimize TAA-loading and MHC-I presentation of WT1 epitopes: increasing translational efficiency or increasing proteasome targeting of the TAA. Codon-optimization of nucleotide sequences is commonly used to enhance expression of a transgene to increase the amount of transgene product, which could be a limiting factor in vaccinations strategies. Algorithms include selection of more commonly used codons to improve translation, but can also include features addressing transcription, mRNA processing and stability as well as protein folding. For the delivery of mRNA, transcription can be excluded as a relevant parameter for improvement, but all others may be useful. It was reported that codon-optimization of the human papillomavirus (HPV) E7 oncoprotein sequence resulted in much higher protein translation and induced CD8+ T cell responses to cryptic epitopes not harbored by wildtype E7 (40). Codon-optimization could, therefore, confer additional advantages then using native mRNA sequences.

Benteyn et al. attempted to optimize translational efficiency of full-length WT1 mRNA (41), but there was no significant advantage of the codon-optimization detected. However, transgene expression was optimized using the pST1 RNA transcription plasmid to generate *in vitro* synthesized mRNA with enhanced translational properties (42). This modification resulted in doubling of the interferon-γ (IFN-γ) responses in a T cell clone. Another feature employed to improve antigen presentation in both MHC-I and MHC-II was the inclusion of endosomal or lysosomal targeting sequences fused to the antigen sequence (43, 44). In particular, the fusion of the C-terminus of LAMP/DC-LAMP to the WT1 mRNA enhanced the IFN-γ also in a T cell clone (41) by increasing both MHC-I presentation and cross-presentation of WT1 peptides. These modifications only require adaptation of the WT1 mRNA sequence, which makes it relatively easy and efficient to implement in a DC vaccine.

Hosoi et al. attempted to optimize proteasome targeting to increase protein degradation and enhance presentation of full-length TAA by triggering co-translational polyubiquitination (45). This triggering of co-translational ubiquitination of the TAA resulted in more efficient priming and expansion of TAA-specific CTLs (45).

To further improve DC vaccination multi-epitope delivery may be beneficial for enhanced CTL activation, e.g., WT1 for AML treatment can be combined with proteinase 3, preferentially expressed antigen in melanoma, telomerase reverse transcriptase, or FLT3-internal tandem duplication (46) for maximal responses. In a multi-epitope vaccine combining multiple myeloma special antigen-1 and Dickkopf-1 to treat multiple myeloma enhanced responses were observed (47).

Viral vectors can also be used to deliver antigen. DCs are highly amenable to lentiviral vector transduction (48). A study using mouse DCs comparing lentiviral vectors that stably integrate into the host genome and provide constant transgene antigen expression to mRNA electroporation showed that lentiviral vector delivery enhanced IFN-γ responses to MAGE-A3 epitopes (49). In the context of UCB-derived DCs, lentiviral vectors could potentially be very useful, since <5 × 10^6^ CD34+ progenitors can be used for the initial transduction and form the basis for expansion of large number of matured DCs (>500 × 10^6^). Another more recent approach uses lentiviral protein transfer vectors for targeting transfer directly into APCs and inducing cytotoxic T cell responses, which could also be used for *ex vivo* delivery (48).

More research is necessary to confirm that the above mentioned modifications could be generally applied to other TAAs or whether this enhances efficacy of CD34-derived DC vaccines.

## Modulating DC Maturation to Improve DC Efficiency

Although consensus is reached that DC vaccines should contain mature rather than immature DCs, there is no consensus about how to polarize and mature DCs to cause optimal anti-tumor responses (50). In 1997, Jonuleit et al. showed that incubation of immature DCs with a cocktail of IL-1β, TNFα, IL-6, and PGE_2_, similar to the GMP-grade available CYTOMIX, resulted in induction of fully matured DCs that seemed to be optimal for generation of IFN-γ producing CD4/CD8+ T cells (51), but very limited efficacy was observed. It is questionable whether to include PGE_2_ as it decreases the expression of IL-12p70 (50), a factor important in induction of tumor-specific Th1 T cells and CTLs facilitating tumor rejection in mouse models (52).

It is reported that DC maturation cocktails containing IFN-γ instead of PGE_2_ [the α-type-1-polarized DC cocktail (αDC1)] increases IL-12p70 levels *in vitro* and *in vivo* boosting TAA-specific CTL levels 40-fold *in vitro* in melanoma (53, 54). Superiority of αDC1-induced maturation was also observed in chronic lymphocytic leukemia assays *in vitro* (55). Similarly, addition of IFN-γ to the CYTOMIX maturation cocktail can increase IL-12p70 production upon CD40 stimulation in WT1 expressing DCs (26).

Another strategy to mature DCs would be to introduce maturation agents with gene therapy. A major advantage of this approach is that DCs can be used within a few hours after delivery of maturation stimuli for vaccination, whereas culturing in maturation agents requires a 24-h incubation period (56). This incubation period *in vitro* potentially leads to DC exhaustion and dampening of the immune response, as shown by Bonehill et al. (56). Single introduction of constitutionally active toll-like receptor 4 (caTLR4) (56, 57) and CD40L (56–58) in immature DCs has shown to induce potent DC maturation, including IL-12p70 production, and both stimuli also act synergistically to superior DC maturation. Melan-A TAA-primed DCs co-electroporated with caTLR4, CD40L, and CD70 mRNA showed an even >200-fold increase in Melan-A specific CTL responses when compared with CYTOMIX matured DCs (56). To date, direct comparisons of this strategy with αDC1-induced maturation of DCs are lacking.

The combination of these three proteins is known as the TriMix strategy, which was developed at the Free University of Brussels, introducing the danger signal caTRL4, the co-stimulatory protein CD40L both to stimulate maturation, and a co-stimulatory protein involved in early T cell activation (CD70) (41, 56, 57, 59, 60). An interesting factor in this strategy is that the DCs mature after electroporation of these factors, eliminating the need of DC incubation with maturation cocktails. A phase-II clinical trial in advanced melanoma showed that combining TriMix-matured moDCs presenting melanoma-associated antigens with ipilumab, an antagonistic CTLA4 antibody, resulted in a 6-month disease control rate of 51%, with an overall tumor response rate of 38% (59). This strategy nicely shows the potential of combining multiple modifications to improve tumor-immunity of DC vaccination.

In the TriMix DCs, maturation of DCs is maximized to improve activation, and polarization of T cells to increase tumor-immunity. However, for an optimal result, it is widely suggested that immunosuppression should be counteracted as well. This is partly established in the TriMix trial by combining the TriMix-matured DCs with ipilimumab, as it inhibits the co-inhibitory effect of the T cell membrane protein CTLA4 on CD80/CD86/CD28 co-stimulation (59).

## Modulating DC Migration to Enhance DC Efficiency

There is no consensus about the most efficient administration route of DC vaccines to migrate to the draining lymph nodes (16). Administration of 111-indium labeled moDCs into patients revealed that less than 5% of the intradermally injected mature moDCs reach the draining lymph nodes (61). A major player in DC migration to the lymph nodes is the C-C motif chemokine receptor 7 (CCR7) (62). Migration to the lymph node is stimulated upon interaction with its ligand, the chemokine C-C motif ligand (CCL21) (63). Adenoviral transduction of DCs with CCR7 (64) and CCL21 (65, 66) showed an ~5.5-fold increase in DC lymph node accumulation, and enhanced tumor rejection and T cell priming in mice *in vivo*, respectively. This could not only increase the effectiveness of the vaccine, but may also reduce the required dose, hence, the efforts and costs associated with vaccine preparation (64). Based on these results, a GMP-grade CCL21 gene-modified monocyte-derived DC vaccine was developed (67), subsequently used in a phase-I clinical trial with non-TAA loaded CCL21 expressing DCs, which triggered TAA-specific T cell responses and enhanced CD8+ T cell tumor infiltration in a subset of patients with non-small cell lung carcinoma (68). Interestingly, CCL21 excretion attracts naive T cells and addition of TAA peptide pools and maturation of DCs may further increase the therapeutic effect. CCL21 could also be applied by mRNA delivery for transient expression similar to adenoviral vectors.

Alternatively, strategies to reduce DC tissue retention could be applied to increase DC migration by disrupting the homing factor E-cadherin (69) (or its positive regulator TGF-β) inducing upregulation of CCR7 (70). Downregulation of E-cadherin upon pro-inflammatory signaling (*via* TNFα, LPS, and IL-1β) further strengthens the hypothesis of involvement of E-cadherin in DC migration (69). The use of small interfering RNAs to downregulate E-cadherin expression on DCs and its effect on migratory function and immune stimulation may be an interesting option. TGF-β is also a known immunosuppressant of DCs, which makes interference of its expression a potential strategy to improve DC vaccination (71).

## MHC-II Cross-Presentation to Enhance DC Function

Major histocompatibility complex-II antigen presentation is required to establish long-term memory anti-tumor immunity through stimulation of CD8+ T cells by CD4+ T cells inducing strong clonal expansion, cytokine production, tumor cell lysis, and T cell memory (72–75). MHC-II knockout DCs were able to generate potent anti-tumor CTL responses *in vivo*, however, without subsequent establishment of a memory anti-tumor response. Therefore, a critical factor in the development of a successful DC vaccine is the ability to present the TAA in both MHC-I and MHC-II context (39).

Full-length TAA mRNA translates into proteins ensuring the presence of MHC-I and MHC-II TAA epitopes, without the requirement of algorithms to predict epitopes per HLA-subtype (14, 39). To further boost this response, a broad TAA peptide pool can be administered in addition to the mRNA electroporation or viral vector delivery. To improve MHC-II presentation of TAA, antigen has also been targeted to endolysosomal compartments to try to improve MHC-II antigen presentation, but this resulted in increased numbers of regulatory T cells (Tregs) and attenuation of tumor immunity (76).

Many studies exploited strategies that link small epitopes to proteins increasing their likelihood of MHC-II presentation (77), however, these epitopes are difficult to predict, are MHC-II restricted, and vary per HLA-subtype and antigen. Therefore, targeting of full-length antigens to the MHC-II pathway is more desirable. Two main MHC-II pathway targeting strategies can be distinguished. The first strategy links the TAA of interest to the cytoplasmic tail of residential endolysosomal proteins, which contains the information for transport to the endolysosomal compartment. Residential endolysosomal proteins tested for this strategy include DC-LAMP (41, 43), LAMP1 (43, 44), and LIMPII (78). The second strategy entails linking of the TAA of interest to the MHC-II associated invariant chain (Ii), a protein important in MHC-II conformational regulation, thereby targeting the TAA to the endolysosomal compartment (43, 79). All studies, irrespective of the endolysosomal protein used, concluded that the increased cross-presentation enhanced CD4+ and CD8+ T cell activation and increased anti-tumor immunity *in vitro* and *in vivo*. Direct comparison of strategies using DC-LAMP, LAMP1, and Ii showed that DC-LAMP and LAMP1 have more pronounced effects than using Ii (43). Interestingly, no clinical studies incorporated these cross-presentation tools into vaccines, even though some cited papers are over 20 years old.

## Modulation of Co-Stimulation to Boost DC Function

A T lymphocyte requires three signals to become fully activated (80), of which co-stimulation is provided by interaction between co-stimulatory molecules expressed on the DC and T lymphocyte. Lack of DC maturation and subsequent co-stimulation induces tolerance against the presented antigen, making these processes of vital importance in the generation of an anti-tumor response. Several co-stimulatory interactions between DCs and T cells have been explored, including CD40/CD40L, 4-1BB/4-1BBL, OX40/OX40L, CD80/86/CD28, CD27/CD70, and GITR/GITRL.

The interaction between CD40 and CD40L, expressed on DCs and T cells respectively, is one of the most potent DC activating signals (56, 81). Modifications to this axis have, therefore, been widely studied to optimize DC vaccination (41, 56–60, 82). Introduction of CD40L into DCs provides autonomous maturation and co-stimulation of DCs (83). In this way, “licensing” of DCs through CD40L interaction with CD4+ Th1 T cells is not required for initiation of a TAA-specific CTL response, and these DCs elicited superior anti-tumor immunity and inhibition of pre-existing tumor growth *via* induction of a TAA-specific CD4+/CD8+ anti-tumor response *in vitro* (56–58, 83) and *in vivo* (41, 82). In addition, introduction of OX40 (84), 4-1BB (85, 86), GITRL (87), and CD70 (41, 56, 57, 59) in DCs is all reported to increase the anti-tumor effect *in vitro* and *in vivo*. All the approaches used mRNA to deliver the co-stimulatory signals.

Upon maturation, OX40L expression is induced in DCs, a ligand of the T-lymphocytic membrane protein OX40. Upregulation of OX40L is stimulated by PGE_2_ (88), but PGE_2_ also downregulates IL-12p70 (50, 52). Therefore, the observed positive effect of OX40/OX40L co-stimulation on tumor rejection (89, 90), through CD4+/CD8+ T cell proliferation, prevention of T cell death, and prevention of tolerance induction, is caused by an unknown mechanism independent of IL-12p70 upregulation (84, 88, 91). Dannull et al. showed that targeting OX40L as a downstream factor of PGE_2_ potentially circumvents the PGE_2_-mediated attenuation of DC function, while utilizing its IL-12p70 independent immunostimulatory capacity in DC vaccination (84).

Another co-stimulatory interaction, 4-1BB/4-1BBL, plays a key role in activation, proliferation, and memory development of CTLs (92). 4-1BB is exploited in second and third generation chimeric antigen receptors in CTLs to provide long-lasting activation potential. 4-1BBL mRNA introduction in HER2/neu TAA expressing DCs resulted in an increased TAA-specific CTL response *in vitro* (85), which was also supported by studies using agonistic anti-4-1BB antibodies *in vitro* and *in vivo* (86, 93). Similar results were observed in the context of HIV-specific T cell responses (86).

A less pronounced effect has been reported for GITR/GITRL co-stimulation, which enhances CD4+/CD8+ T cell responses, while inhibiting Treg-mediated immune suppression (87, 94). A new approach to introduce heavy and light chains of an agonistic anti-GITR antibody in DCs could stimulate this pathway (87). Combining vaccination of these anti-GITR-secreting DCs with TAA-presenting DCs resulted in an increased CTL response, and inhibition of sensitivity to Treg mediated immune suppression, thereby increasing anti-tumor immunity *in vitro* and *in vivo*. This approach may cause less systemic adverse effects, while maintaining the anti-tumor response (87).

Finally, CD27/CD70 interaction promotes clonal expansion of primed CD4+/CD8+ T cells, mostly *via* supporting survival of primed T cell clones (87). The constitutive expression of CD27 on T cells, by contrast to the other T-lymphocytic co-stimulatory molecules, indicates an important role during early T cell priming, making its ligand an interesting molecule to modify in DC vaccination. Keller et al. showed that constitutive expression of CD70 in steady-state immature DCs loaded with TAA can overcome peripheral resistance (95), and resulted in a robust effector and memory CTL response *in vitro* and *in vivo*, even in absence of CD4+ T cells (96).

Multiple papers reported the beneficial effects of combining autonomous DC maturation *via* CD40L introduction with factors enhancing T cell activation through 4-1BBL (86) and CD70, in combination with caTRL4 (41, 56, 57, 59, 60), respectively, on tumor immunity. Introduction of these co-stimulatory provide multiple opportunities to enhance tumor immunity through incorporation into DC vaccines.

## Interferance with Co-Inhibitory and Immunosuppressive Pathways to Enhance DC Function

Dendritic cells should live long enough to generate a potent anti-tumor response, but have a physiological short lifespan (14). Moreover, remaining activatory DCs presenting TAA in MHC-I context are killed by activated TAA-specific CTLs, which probably also is a physiological mechanism to prevent exaggeration of immune responses (97–99). A major concern in DC vaccination is that DC injection in TAA-primed mice results in DC elimination before reaching the draining lymph node (100, 101). DC elimination by CTLs can even be used as a measure for effective cytotoxic response (100, 101). DC apoptosis is triggered physiologically, as well as by the tumor microenvironment. Inhibiting DC apoptosis can prolong the DC lifespan after siRNA-mediated silencing of the pro-apoptotic proteins BAK/BAX (97, 102), BIM (98), and PTEN (99) *in vitro* and *in vivo*, which all resulted in more efficient TAA-specific CTL responses. Disadvantages inherent to modifications of pro-apoptotic proteins are the potential oncogenicity, restricting its use to temporary silencing strategies, e.g., siRNAs.

A second strategy is to inhibit tolerogenic DC development to prevent induction of anergic T cells. Silencing of several factors has been proposed, including suppressor of cytokine signaling 1 (SOCS1), IL-10, IL-10R, and TGF-βR. SOCS1 is an inducible negative feedback inhibitor of the JAK/STAT pathway and thereby negatively regulates expression of multiple cytokines, including IFN-γ, IL-2, IL-6, IL-7, IL-12, and IL-15 (103). SOCS1 deficient DCs are reported to be extremely hyperresponsive to IL-4 and IFN-γ and cause abnormal accumulation of antigen-specific T cells (104). Vaccination with HPV16mE7 pulsed, shRNA-mediated SOCS1-silenced DCs showed significantly improved anti-tumor effects compared to non-SOCS1-silenced controls *in vitro* and *in vivo* (103).

The most well-known immunosuppressive cytokines are IL-10 and TGF-β, produced by Tregs, among others, to induce DC tolerance and anergic T cells (105). The fact that high serum levels of both IL-10 and TGF-β are correlated with poor prognosis in several types of cancer indicates an interesting role of inhibition of their expression or responsiveness to their presence (71, 106). As IL-10 can be produced by DCs (107), one way to decrease its effect is to silence IL-10 expression by DCs. However, as IL-10 is also produced by other cell sources, it is probably more effective to knockout its receptor, IL-10R (106, 108), or a combination of both (109). Both studies evidently report benefits on DC maturation and anti-tumor effects *in vitro* and *in vivo* and suggest that clinical translation of this will greatly enhance DC vaccination potency. A similar effect was observed TGF-β receptor (TGF-βR) was silenced (71, 108). Ahn et al. tested the individual as well as the combined potency of siRNA-mediated silencing of IL-10R, TGF-βR, PTEN, and BIM (108). IL-10R silencing initiated the strongest individual CTL response, followed by TGF-βR. Furthermore, a cocktail combining IL-10R and TGF-βR siRNAs generated the strongest overall CTL response *in vitro* and *in vivo*.

A third strategy aims to decrease DC-mediated T cell apoptosis through co-inhibitory signals, e.g., programmed cell death 1 (PD-1) interaction with its ligand (PD-L1), which is widely described as one of the most potent immunoinhibitory interactions (110). PD-1/PD-L1 interaction is known to inhibit T cell proliferation, survival and effector function, induces apoptosis of tumor-specific T cells, and promotes Treg differentiation as well as resistance of tumor cells to CTL attacks (111). PD-1 expressing TAA-specific T cell function is inhibited by tumor PD-L1 expression, as well as by tumor-induced PD-L1 expression of DCs (111). Advanced clinical trials with PD-1 and PD-L1 antibodies show very promising results in preventing axis signaling in non-small cell lung cancer, indicating the potential that blocking this pathway enhances anti-tumor immunity (112). Silencing of PD-L1, on its own (113) or in combination with its phagocyte-restricted relative PD-L2 (114), shows augmented *ex vivo* TAA-specific CTL responses, which is also confirmed *in vivo* (115). Moreover, combined silencing of PD-L1 and IL-10 in DC vaccination showed even stronger induction of anti-tumor responses *in vitro* and *in vivo* (106) indicating the potential of combining DC modifications in maximizing anti-tumor responses.

Next to PD-1/PD-L1, indoleamine 2,3-dioxygenase (IDO) could have similar effects on T lymphocytes. IDO can be secreted by DCs and depletes the microenvironment from tryptophan, an essential amino acid required for T cell proliferation and survival (116). Furthermore, various tryptophan metabolites are directly immunosuppressive to T cells. Moreover, IDO-expressing cells are able to differentiate naive T cells into Treg cells, thereby further suppressing anti-tumor immunity. The ability to produce IDO depends on the DC subset, and signals present in the tumor microenvironment that can contribute to the amount of IDO produced (117). IDO upregulation was clearly shown in DCs used for vaccination 24 h after maturation in melanoma patients, indicating the potential relevance of IDO silencing (116). Several studies have indicated decreased tumor sizes, reduced CD4+/CD8+ T cell apoptosis, enhanced T cell proliferation and CTL activity, and decreased Treg cell numbers upon IDO silencing, which was confirmed *in vitro, in vivo* as well as in patient studies (116, 118).

Nonetheless, immunosuppression of the DCs by factors like IL-10, TGF-β, PD-1, and IDO is ignored in many studies, including the TriMix trial, and may cause substantial downregulation of the anti-tumor response. Especially IL-10 and PD-1 are widely reported to be important inhibitors of immune responses, making these proteins (or their ligands) interesting targets to silence. Silencing of PD-L1 in DCs is expected to cause T cell priming and activation. In the case of solid tumors, PD-L1 is often also expressed by the tumor itself, and may locally provide inhibitory signals affecting these primed T cells. In that way, effector T lymphocyte function can still be inhibited by PD-L1 binding to the T cell membrane protein PD-1. However, the remaining tumor burden in most treated AML patients is relatively low; hence, this might turn out to be less of an issue in AML therapy. The application of DC vaccine delivery during the early stages of immune reconstitution may significantly induce priming to eliminate residual AML blasts effectively. It is expected that the generation of DCs from a UCB will take approximately 4 weeks to generate. Thereafter, the initial DC vaccine can be infused into the patient, followed by multiple DC injections to further boost anti-tumor responses of *de novo* generated T cells. In the future, an interesting strategy might be to add TAA-specific PD-1 knockout effector T cells to the DC vaccine as well, thereby potentially stimulating and expanding these gene-modified T cells to boost anti-tumor responses. A head-to-head comparison of silencing strategies of these proteins in CD34-derived DCs is needed to select the most promising to overcome immunosuppression. However, the use of siRNA in this application may not be as effective as techniques to permanently eliminate expression, because these cells are heavily replicating.

An overview of the numerous modifications tested on DCs is summarized in Table 1.

**Table 1:** Dendritic cell (DC) modifications to enhance anti-tumor induced immunity.

Process	Modification	*In Vitro* studies	*In Vivo* studies	Clinical studies	Reference
Major histocompatibility complex (MHC)-I presentation	↑ Translational efficiency^a^	↑ IFN-γ production	↑ Tumor-associated antigen (TAA)-specific cytotoxic T lymphocyte (CTL) response	N.A.	(41)
			↑ Anti-tumor response		

	Ubiquitin addition to mRNA	↑ CTL expansion	↑ TAA-specific CTL response	N.A.	(45)
		↑ Proteosome targeting			
			↑ IFN-γ production		
		↑ IFN-γ production			

DC maturation	caTRL4 introduction	↑ Interleukin (IL)-12p70	N.A.	X Objective responses+ IFN-α-2β: partial response and stable disease + ipilimumab: 51% 6-month disease control rate^b^	(56, 57, 60)
		↑ CD4+ and CTL expansion			
		↑ IFN-γ and TNF-α production			
		↑ CTL cytolytic activity			

	CD40L introduction	IL-12p70	↑ Anti-tumor response	X Objective responses+ IFN-α-2β: partial response and stable disease + ipilimumab: 51% 6-month disease control rate^b^	(41, 56–60)
		↑ CD4+ and CTL expansion			
		↑ IFN-γ and TNF-α production	↑ CD4+ and CTL tumor Infiltration		
		↑ CTL cytolytic Activity			

DC migration	C-C motif chemokine receptor 7 introduction	↑ Chemotactic activity	↑ Chemotactic activity	N.A.	(64)
		↑ CD40 and CD86 expression			
			↑ Anti-tumor response		
		↑ Anti-tumor response			

	chemokine C-C motif ligand introduction	↑ Chemotactic activity	↑ DC and T cell at tumor site	↑ CTL tumor infiltration	(65, 66)
				Induction of TAA-specific responses in a subset of patients in NSCLC	
			↑ Anti-tumor response		
			↑ IFN-γ and IL-12 production		

	E-cadherin downregulation^c^	N.A.	N.A.	N.A.	N.A.

Cross-presentation	C-terminal tail addition of DC-LAMP/LAMP1/LIMPII	↑ CD4+ and CTL expansion	↑ Anti-tumor immunity	N.A.	DC-LAMP: (41, 43, 119) LAMP1: (43, 44) LIMPII: (78)
		↑ IFN-γ production			

	Linking to MHC-II associated invariant chain	↑ CD4+ and CTL expansion	↑ Anti-tumor immunity	N.A.	(43, 79)
		↑ IFN-γ production			

Co-stimulation	CD40L introduction	*See DC maturation*	*See DC maturation*	*See DC maturation*	*See DC maturation*

	OX40L introduction	↑ CD4+ and CTL expansion	↑ Anti-tumor immunity	N.A.	(84)
		↑ DC migration			
		=IL-12p70			
		Th1 T cell polarization			

	4-1BBL introduction	↑ CD40 and CD86 expression	N.A.	N.A.	(85, 86)
		↑ CTL expansion and activity			
		↓ Treg activity			

	Anti-GITR introduction	↓ Treg activity	↑ Anti-tumor immunity	N.A.	(87)
		↑ Treg suppression			
			Long-term memory responses		
			↑ CD4+ and CTL expansion		
			↑ Treg expansion		

	CD70	↑ CTL expansion	↑ CTL expansion	X Objective responses+ IFN-α-2β: partial response and stable disease in melanoma + ipilimumab: 51% 6-month disease control rate in melanoma^b^	(41, 56, 57, 59, 60, 96)
		↑ CTL memory	↑ CTL memory		
		↑ IFN-γ production			
			↑ Anti-tumor response		

Immunosuppression	↓ Apoptosis^d^	↑ Resistance to CTL killing	↑ Anti-tumor response	N.A.	(97–99, 102)
		↑ CTL expansion			
			↑ DC survival		
		↑ IFN-γ production			

	Suppressor of cytokine signaling 1 downregulation	↑ DC maturation	↑ CTL cytolytic Activity	N.A.	(103)
		↑ IL-12p70, TNF-α			
			↑ Anti-tumor response		
	
	IL-10(R) downregulation	↑ MHC-II and CD40 expression	↑ Anti-tumor response	N.A.	(106–109)
		↑ IL-12p70			
		↑ Anti-tumor response	↑ CTL expansion		
		↑ CTL expansion			
	
	TGF-βR downregulation	↑ CD80/86 expression	↑ Anti-tumor response	N.A.	(71, 108)
			↑ IFN-γ and IL-12p70		
			↑ CTL expansion		
	
	PD-L1 downregulation	↑ IL-12p70	↑ CTL expansion	N.A.	(113, 115)
		↓ IL-10 secretion			
		↑ Anti-tumor response			
		↑ CTL expansion			
	
	Indoleamine 2,3-dioxygenase downregulation	=DC maturation	↓ T cell apoptosis	+Ipilimumab: ↓ size of melanoma metastases in subset of patients	(116, 118, 120, 121)
			↑ CTL expansion		
		↑ CD4+ and CTL expansion	↑ CTL cytolytic activity		
			↓ Treg expansion and activity		

*^a^Removal nuclear localization signal, in silico mRNA optimization for optimal codon usage and G/C content, removal of splice sites, and subcloning in in vitro transcription pST1 vector*.

*^b^Only tested in the Trimix combination: caTRL4, CD40L, and CD70*.

*^c^Hypothesis, not supported by clinical DC vaccine studies yet*.

*^d^Through introduction of BAK/BAX, BIM, or PTEN*.

## Novel Techniques to Modify CD34-Derived DCs to Potentially Improve Potency

Numerous phase-I DC vaccination-based clinical trials have confirmed the safety of using immature, mature, and TAA-expressing DC vaccines (15, 17). The use of mRNA is relatively safe, because of the temporary expression of the antigen, DC maturation signal or co-stimulatory domain. However, expression cannot be restricted to certain cell types if that is required. Integrating viral vectors may provide longer expression of molecules of interest, but has the risk to potentially cause upregulation of proto-oncogenes (122). Since DCs are generally short-lived this risk may be minimal if the genetic alterations are applied close to application into the patient. Risks of insertional oncogenesis may be increased if CD34+ progenitors are genetically altered before expansion, differentiation, and maturation, particularly because these cells are actively dividing.

The use of siRNAs in DC vaccines is promising, and has shown potential use to reduce expression of co-inhibitory signals in moDCs. Efficiency in UCB-derived DCs has not been shown yet, but may be hampered by the loss of inhibitory ability of siRNAs in cycling cells. This may require precise fine-tuning of delivery of the siRNAs to obtain effective reduction of genes of interest.

The CD34+ expansion phase of the two-step protocol (26) provides a unique environment to modify the DCs to enhance treatment efficiency. However, it is important to carefully select the factors to be removed or introduced in this phase, as this might induce differentiation or decreased proliferation of CD34+ HSPCs. Gene-editing tools to permanently eliminate expression have been used for more than a decade. Initially, zinc finger nucleases were genome sequence specific with relatively low efficiency and toxicities in hematopoietic cells. Gene-editing tools that cause insertions and deletions on a genomic level have not been applied to DCs. These techniques have been mainly used on T cells. In particular, Transcription activator-like effector nucleases mediated gene-editing and clustered regularly interspaced short palindromic repeats (CRISPR)/Cas9 system are used to eliminate expression of the T cell receptor or co-inhibitory signal PD-1 (123).

More recently, Gundry et al. showed efficient knockout of genes in CD34+ HSPCs (~75%) by CRISPR/Cas9 (46). They show that their strategy to electroporate CD34+ HSPCs with Cas9/sgRNA ribonucleoprotein (RNP) complexes causes efficient CRISPR/Cas9 targeting of up to four sgRNAs. No major effects of gene-editing were observed on viability and proliferation capacity. On the other hand, CRISPR/Cas9-mediated targeting of multiple targets at the same time might cause genomic translocations (124), which are potentially cytotoxic.

This strategy permits for efficient knockout of one, two, or more factors involved in immunosuppression (e.g., PD-L1, IL-10, IL-10R, TGF-β, TGF-βR, and IDO). Strikingly, Gundry et al. also reported that this method allows for efficient homology-directed repair gene-editing (125). In this way, an expression cassette containing WT1 cDNA could even be integrated at one of the gene-editing target sites, allowing for constitutive WT1 expression. Combining the TriMix strategy with gene-edited DCs could potentially be a very potent combination.

The recent advances of CRISPR/Cas9 gene-editing in CD34+ HSPCs make this strategy more efficient, commonly applicable, and technically feasible to include in DC vaccines. If the co-inhibitory genes do not affect cell-cycling or viability, gene-editing tools could be very valuable in creating more potent off-the-shelf CB-derived DC vaccines.

The major risks of CRISPR/Cas9 gene-editing are the potential off-target genome cleavage sites it can create (124). Fu et al. showed that off-target effects can be observed at sites that differ by five nucleotides from on-target sequences, indicating that this might cause efficient gene-editing of off-target sites in CRISPR/Cas9 modified cells (124). CD34+ HSPCs are highly proliferative cells and off-target cleavage might promote tumorigenesis. This risk can be reduced by using Cas9-gRNA RNP complexes rather than using mRNA or plasmids to deliver Cas9, thereby limiting their time-frame of action. Kleinstiver et al. showed that mutating four Cas9 amino acids important in DNA binding energy almost completely diminishes the off-target risk of CRISPR/Cas9, while maintaining its on-target effect (126). This indicates that switching to this mutated version of Cas9 (spCas9-HF1) will potentially further increase CRISPR/Cas9 safety.

It is important to note that, even though studies report extremely low incidences of off-target mutations with wildtype Cas9 in CD34+ HSPCs (127), more research is required to develop accurate off-target site prediction tools. Many studies report low off-target effects, but based on this *in silico* predicted off-target sites rather than on whole-genome sequencing. Hence, whole-genome sequencing of gene-edited cells should be performed to improve the off-target prediction algorithms.

It is also reported that multiplexing CRISPR/Cas9-mediated gene-editing targeting more than one target may result in genomic translocations (128). Poirot et al. performed CRISPR/Cas9 gene-editing in duplex and reported a translocation frequency ranging from 10^−4^ to 2 × 10^−2^ (128). After 38 days of culturing translocation frequencies remained stable or reduced, indicating that these translocations are safe and did not cause proliferative advantages. It is very important to assess the translocation frequency and the consequence of these translocations per specific gRNA sequence and the downstream effects on highly proliferative CD34+ HSPCs, and whether this may cause gene-edited related tumorigenicity is yet unknown.

When evaluating the *in vivo* mouse studies performed to assess DC vaccination, two main strategies could be distinguished. Most studies test the DC vaccines on a complete murine background (wildtype mice, with murine tumors and murine DCs) (87, 96, 120). Inherent to these mouse studies is that translation can be difficult due to interspecies differences. Another option is the use of humanized mouse models, e.g., NOD/SCID or more severe immune compromised NOD/SCID gamma mice that allow introduction of human DCs, TAA-specific CD8+ T cells, and human tumor cells (115). However, this also has its limitations, as these models lack the presence of interaction with human immune cells that could contribute to tumor immunity. The translation of gene-modified DCs to clinical application could be improved by the use suitable mouse models with the humanized immune systems (129).

To summarize, DC vaccination has a proven track-record of safety, but addition of genetic modifications could introduce some safety concerns that need to be addressed. The short lifespan of DCs to generate tumor immunity should improve safety of using these cells, which reduces the likelihood that DCs acquire the ability to divide uncontrollably.

## An Off-The-Shelf DC Vaccination Approach

CD34-derived DC can be used as a basis to develop personalized cellular vaccines. This strategy is very promising in combination with UCB transplantation. By using the same UCB-unit the risk of adverse effects is significantly decreased by preventing mismatching. Nonetheless, the personalized nature makes this strategy laborious, relatively expensive and requires automated systems to obtain consistent high quality products. The generation of an off-the-shelf product could make this approach more cost-effective and potentially more suitable for standardization for multicentre trials.

Off-the-shelf DC vaccination products are still in their infancy, and more research and technical advances are needed to be able to generate more effective gene therapy products that have a proven quality ready for infusion into cancer patients.

## Conclusion and Outlook

The sporadically observed clinical responses indicate the necessity to improve DC vaccinations. Literature suggests that intervening in early DC maturation and activation can cause a cascade-like reaction that eventually also improves downstream activatory processes. It is also widely reported that the immunosuppressive tumor microenvironment is still able to downregulate the most potently activated DCs. Hence, combining modifications of early DC activation processes, such as caTRL4, CD40L, CD70 with elimination of immunosuppressive signaling, such as IL-10R and PD-L1, may drive optimal anti-tumor T cell responses by maximizing both co-stimulatory/co-inhibitory ends of the spectrum.

Tumor-associated antigens can be delivered by optimized mRNA sequences for efficient processing and MHC-I and MHC-II presentation, which could incorporate DC-LAMP C-terminal sequences, ubiquitination or mRNA transcription from optimized transcription vectors to be electroporated in CD34+ derived DCs.

The use of CD34+ HSPCs to generate UCB-derived DCs provides an opportunity during the expansion/differentiation phase to manufacture gene-modified cellular products. Recent progress using state-of-the-art gene therapy vectors, such as self-inactivating third generation lentiviral vectors, that are used in clinical trials to treat inherited diseases and in T cell immunotherapies to treat cancer, have shown the ability to transduce hematopoietic stem cell progenitors effectively, as well as provided evidence for long-term safety. Application to DC vaccines may provide advantageous effects compared with using mRNA. Together with the recent progress in CRISPR/Cas9-mediated gene-editing efficiency of CD34+ HSPCs, this provides a unique cell pool to knockout immunosuppressive factors. The small number of CD34+ HSPCs may aid to reduce the viral vector batches and gene-editing tools required before expansion, differentiation, and maturation. It is important to investigate any negative effects on these phases during DC development.

There is a clear need for consistent comparative studies to compare DC subsets, maturation strategies, and modifications. Although many modifications have been tested in laboratory/pre-clinical studies and resulted in improved efficiency *in vitro* and *in vivo* models, very few of these modifications have translated into clinical applications. The use of state-of-the-art gene therapy vectors and gene-editing tools may create opportunities for next generation therapies with high efficacy for treating hematologic cancers and solid tumors.

## Author Contributions

AC and NT wrote the manuscript. JB and SN made substantial and intellectual contributions to the work and approved it for publication.

## Conflict of Interest Statement

The authors declare that the research was conducted in the absence of any commercial or financial relationships that could be construed as a potential conflict of interest.
